# *CLD1* Reverses the Ubiquinone Insufficiency of Mutant *cat5/coq7* in a *Saccharomyces cerevisiae* Model System

**DOI:** 10.1371/journal.pone.0162165

**Published:** 2016-09-07

**Authors:** Adwitiya Kar, Haley Beam, Megan B. Borror, Michael Luckow, Xiaoli Gao, Shane L. Rea

**Affiliations:** 1 Barshop Institute for Longevity and Aging Studies, University of Texas Health Science Center at San Antonio, San Antonio, Texas, United States of America; 2 Department of Physiology University of Texas Health Science Center at San Antonio, San Antonio, Texas, United States of America; 3 Institute for Behavioral Genetics, University of Colorado at Boulder, Colorado, United States of America; 4 Department of Biochemistry, University of Texas Health Science Center at San Antonio, San Antonio, Texas, United States of America; Texas A&M University, UNITED STATES

## Abstract

Ubiquinone (Q_n_) functions as a mobile electron carrier in mitochondria. In humans, Q biosynthetic pathway mutations lead to Q_10_ deficiency, a life threatening disorder. We have used a *Saccharomyces cerevisiae* model of Q_6_ deficiency to screen for new modulators of ubiquinone biosynthesis. We generated several hypomorphic alleles of *coq7/cat5 (clk-1* in *Caenorhabditis elegans*) encoding the penultimate enzyme in Q biosynthesis which converts 5-demethoxy Q_6_ (DMQ_6_) to 5-demethyl Q_6_, and screened for genes that, when overexpressed, suppressed their inability to grow on non-fermentable ethanol—implying recovery of lost mitochondrial function. Through this approach we identified Cardiolipin-specific Deacylase 1 (*CLD1)*, a gene encoding a phospholipase A_2_ required for cardiolipin acyl remodeling. Interestingly, not all *coq7* mutants were suppressed by Cld1p overexpression, and molecular modeling of the mutant Coq7p proteins that were suppressed showed they all contained disruptions in a hydrophobic α-helix that is predicted to mediate membrane-binding. *CLD1* overexpression in the suppressible *coq7* mutants restored the ratio of DMQ_6_ to Q_6_ toward wild type levels, suggesting recovery of lost Coq7p function. Identification of a spontaneous Cld1p loss-of-function mutation illustrated that Cld1p activity was required for *coq7* suppression. This observation was further supported by HPLC-ESI-MS/MS profiling of monolysocardiolipin, the product of Cld1p. In summary, our results present a novel example of a lipid remodeling enzyme reversing a mitochondrial ubiquinone insufficiency by facilitating recovery of hypomorphic enzymatic function.

## Introduction

Coenzyme Q, or ubiquinone (Q), is a redox-active biomolecule best known for its role as a mobile electron carrier in the mitochondrial electron transport chain (ETC). Q is comprised of a functionalized benzoquinone head group and a polyisoprenoid tail, the length of which is species-specific—ten isoprenoid units in humans (Q_10_), nine in *Caenorhabditis elegans* (Q_9_), and six in *Saccharomyces cerevisiae* (Q_6_). Primary Q_10_ deficiency manifests clinically as a collection of heterogeneous diseases that depend on the severity of Q_10_ loss, and include encephalomyopathy, severe infantile multisystemic disease, cerebellar ataxia, Leigh syndrome, and isolated myopathy [1–4]. Presently, disruption of nine genes has been linked to primary Q_10_ deficiency in humans: *ADCK3/COQ* and its paralog *ADCK4*, *COQ2*, *COQ4*, *COQ6*, *COQ7*, *COQ*9, *PDSS1/COQ1* and *PDSS2* [5, 6]. Genetic studies in mice show that complete removal of either *Coq3*, *Coq4*, *Coq7* or *Pdss2* results in embryonic lethality [7], and it is likely that complete loss of these genes in humans is also lethal.

Extensive work over the past two decades has shed light on the pathways involved in Q biosynthesis in cells [5]. Much of this work has exploited *S*. *cerevisiae* and there is considerable overlap with humans. Under aerobic conditions, *S*. *cerevisiae* preferentially ferments glucose to ethanol. When glucose becomes exhausted (at the diauxic shift), or when cells are cultured on a non-fermentable carbon source such as ethanol, *S*. *cerevisiae* becomes obliged to use its mitochondrial ETC machinery and hence Q production becomes essential. The ability of *S*. *cerevisiae* to shuttle between two metabolic states, coupled with the capacity to survive in either a haploid or a diploid form, has resulted in the identification of at least nine genes (*COQ1* to *COQ9*) that are essential for the biosynthesis of Q in this species [8]. Orthologs of all nine genes are found in mammals [9]. Additional genes are also known to be involved in the manufacture of Q in *S*. *cerevisiae*: for example, a biosynthetic sub-pathway involving *p*-amino benzoic acid has been described [10, 11]. More genes likely await identification [5, 12].

In yeast, Q biosynthesis begins with Coq1p and Coq2p; these two enzymes co-operate to form 4-hydroxy-3-hexaprenyl benzoate (HHB). A 700 kDa ‘pre-complex’, comprised of Coq3p, Coq4p, Coq6p and Coq9p, which is bound to the inner mitochondrial membrane, next modifies the benzoate head group of HHB to form 5-demethoxy Q_6_ (DMQ_6_) [13, 14]. This compound is the penultimate intermediate in Q biosynthesis and completion of Q_6_ formation occurs when yeast transition toward the diauxic shift, at which point the 700 kDa pre-complex matures into a 1.3 MDa complex following the regulated recruitment of Coq7p [14, 15]. Coq7p directly binds to Coq9p [16, 17] and this event may be regulated by the Coq7p phosphatase, Ptc7p [18]. Clarke and colleagues have reported that complete removal of Coq7p in *coq7* null mutants results only in HHB formation [19], while loss-of-function *coq7* point mutants, such as G65D and E194K, accumulate DMQ_6_ [20]. This suggests that Coq7p may in fact be a constitutive component of the Q biosynthetic complex that is held in an inactive state until required. Modeling studies clearly show that Coq7p is a DMQ_6_ hydroxylase [21], and both modeling and experimental studies show Coq7p is a peripheral-membrane bound protein [22, 23]. Coq7p might therefore toggle between strongly- and weakly membrane-bound states which in turn determine both its final activity and its ability to be detected in the soluble 700 kDa pre-complex. Consistent with this notion, overexpression of the Coq7p kinase, Coq8p [24], stabilizes the 700kDa pre-complex in *coq7* null mutants and re-permits Q_6_ assembly all the way to DMQ_6_ [14].

In the nematode *C*. *elegans*, *clk-1/coq7* null mutants are unexpectedly viable [25]. Although respiration is impaired in these animals [26], and they are slow-growing and behaviorally-sluggish, more surprisingly they are long-lived [25]. Part of this ability to survive under circumstances when other species cannot is now known to be due to the ability of worms to extract Q_8_ from their *E*. *coli* food supply. Nonetheless, mutant *clk-1* worms cultured for several generations on a bacterial food source that is unable to manufacture Q_8_ (GD1 *E*. *coli*) remain phenotypically long-lived [27]. Under these conditions, otherwise wild type worms also display life-extension. Expression profiling reveals that these animals elicit a transcriptional response similar to the retrograde response activated following mitochondrial ETC disruption in petite yeast, which are also long lived [28]. Moreover, *clk-1* mutants reprogram their metabolism, similar to other long-lived mitochondrial electron transport chain mutants in *C*. *elegans*, including *nuo-6(qm200)* and *isp-1(qm150)—*which disrupt complex I and III, respectively [29, 30]. At least part of the *clk-1* longevity response is now known to be due to a nuclear-targeted form of CLK-1 that unexpectedly binds chromatin [31].

Given the essential nature of *COQ7* in humans, its centrality to Q production in cells, and its unexpected role in lifespan control of *C*. *elegans*, we sought to identify new genetic loci that could suppress the disruption of this gene in *S*. *cerevisiae*. To this end we identify the cardiolipin remodeling enzyme CardioLipin-specific Deacylase 1 (Cld1p) as a novel modulator of Coq7p activity.

## Results

In an effort to identify genes that are able to overcome the essential requirement of Coq7p in yeast cultured on a non-fermentable carbon source, we transformed a *coq7* null mutant *(*Δ*coq7)* with a library of genomic DNA fragments isolated from wild type yeast and contained on a high copy number vector. Transformants were directly selected for growth on ethanol (YEPE_3%_). We obtained no suppressors, which was unexpected since PCR confirmed that our library contained multiple copies of the wild type *COQ7* locus. If Δ*coq7* cells containing a *bona fide* copy of *COQ7* were allowed to exhaust their supply of glucose prior to selection on ethanol, however, then could growth be rescued. This observation suggests that initiation of the diauxic shift in yeast is required to de-repress a genetic network permissive for *COQ7* expression and activation. This idea is consistent with previous reports showing (i) Coq7p is dephosphorylated upon entry into the diauxic shift [18, 32], (ii) Q_6_ biosynthesis proceeds only to the level of DMQ_6_ prior to the diauxic shift [14], (iii) DMQ_6_ does not support respiration in yeast [33], and (iv) Coq7p is part of a ubiquinone-biosynthesis megacomplex, the stability of which is dependent upon full length Coq7p [14, 15]. We reasoned that many prior studies that searched for *coq7* loss-of-function suppressor genes using a similar library-screening approach may have missed targets either because cultures had not been at the diauxic shift prior to transformation and selection, or because yeast cells have an absolute requirement for small amounts of Coq7p, or both. In an effort to identify novel *coq7* suppressors, and to by-pass both of these potential limitations, we first generated a novel *coq7* allelic series, comprised of multiple hypomorphic mutants, and then used it to undertake high-copy genomic DNA suppressor screens. We also made use of selection media comprised of non-fermentable ethanol supplemented with a small amount of dextrose (YEPE_3% + 0.1% DEX._) in order to facilitate isolation of potential suppressors; our rational being that small amounts of glucose would provide a smooth transition into the diauxic shift after transformation.

### Generation of a *coq7* allelic series

**Fig 1:**
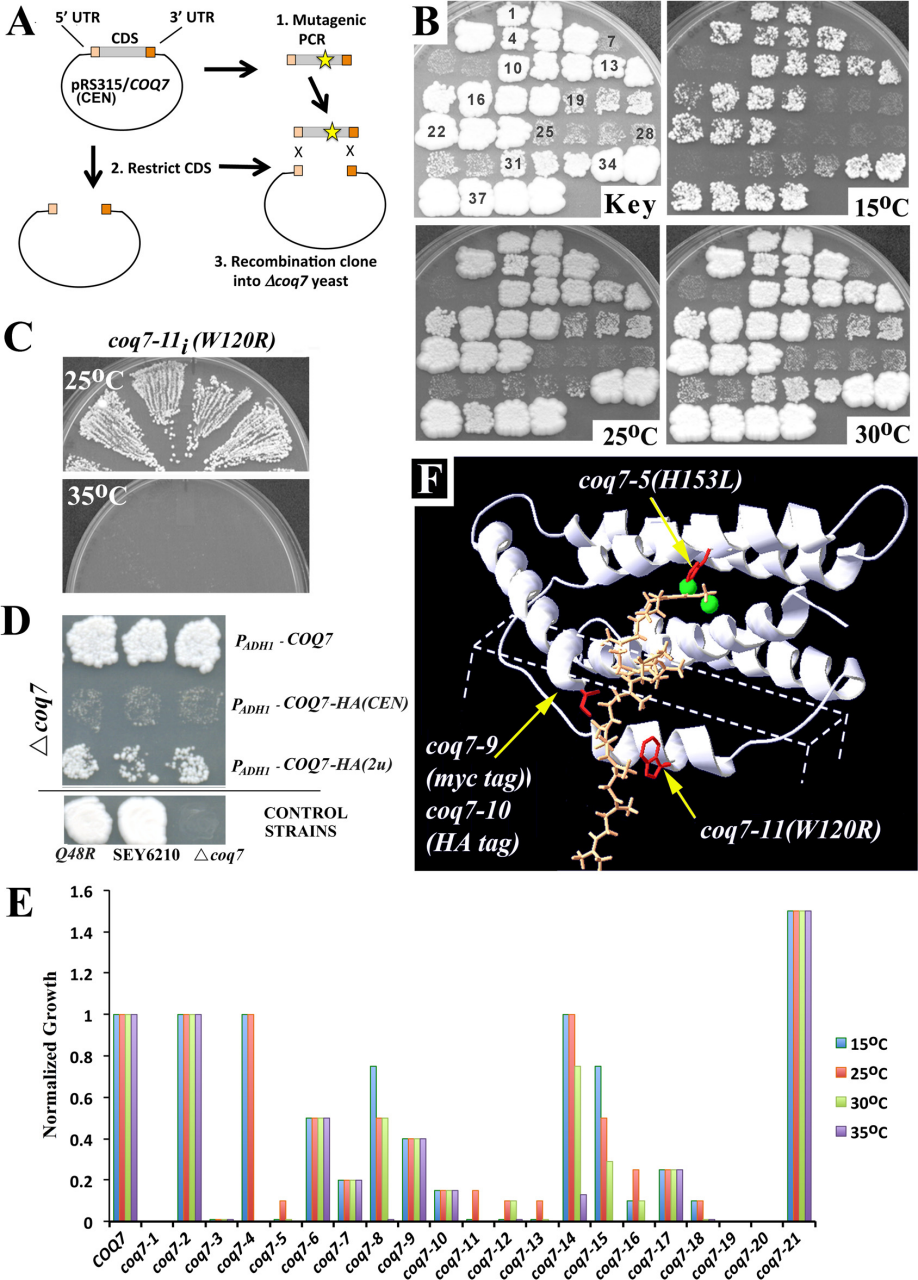
Generation and Characterization of a *coq7* Hypomorphic Allelic Series. **(A)** Strategy for generating mutant *coq7* alleles: *coq7-19(Δcoq7)* yeast were transformed with a mix of PCR mutagenized *coq7* constructs and then hypomorphic alleles identified by slow growth on 3% ethanol (YEPE_3%_). **(B)** Mutagenized clones were patched in triplicate, then replica plated onto YEPE_3%_, and monitored for growth at 15°C, 25°C and 30°C (*top left panel* shows patch numbering) relative to wild type SEY6210 yeast (patches #37–39). Alleles of interest are described in full in S1 Table and include: *coq7-6* (patches # 13–15); *coq7-11* (# 19–21); *coq7-13* (# 25–27); *coq7-5* (# 28–30); *coq7-16* (# 31–33) and *coq7-2(Q48R)* (# 34–36). **(C)** The *coq7-11* allele is temperature-sensitive and inviable at 35°C when integrated back into the wild type *COQ7* locus. Shown are four independent re-integrants. **(D)** Addition of a *HA* or *myc* epitope tag to the C-terminus of wild type Coq7p results in hypomorphic growth on YEPE_3%_. Shown also is the effect of *COQ7* copy number (CEN—*low* and 2μ- *high*) and promoter identity (*ADH1* vs. native) on growth at 30°C YEPE_3%_ (10 days). *P*_*ADH1*_*-COQ7-HA(CEN)* was used for the library screen described in Fig 2. *coq7-2(Q48R)* grows indistinguishably from wild type at 30°C. **(E)** Relative growth rate of mutant *coq7* alleles versus wild type yeast (SEY6210) cultured at four different temperatures– 15°C, 25°C, 30°C and 35°C (refer to Table 1 for allele identification, and S1 Table for raw data). Data is normalized to SEY6210 cell density at late log phase, for each respective temperature. Data for *coq7-5*, *coq7-15* and *coq7-16* at 35°C was not collected. **(F)** Location of relevant amino acid disruptions caused by various hypomorphic *coq7* alleles *(labeled)*. Changes affect highly conserved residues *(red)* and have been mapped onto a model of monomeric rat CLK-1/Coq7p [21]. Shown is the predicted position of the conserved C-terminus submerged in the mitochondrial inner membrane (*dotted box*), the di-iron-containing active site (green) and the DMQ_6_ substrate loaded into the active site (saffron). Coq7 dimerizes [35] and we have previously provided a model of dimeric rat CLK-1 [21].

Mutagenic PCR was used to generate over seventy *coq7* alleles that were hypomorphic for growth on YEPE_3%_ (Fig 1A and 1B). All mutant alleles generated using this approach carried a Q48R point mutation that was present in the parent allele before mutagenesis. *coq7-2(Q48R)* yeast grow indistinguishably from wild type SEY6210 yeast between the biologically relevant temperatures of 15–35°C. We have named this allele *coq7-2* to distinguish it from the *COQ7* sequence of SEY6210. Alleles that were selected for further study are summarized in Table 1 and described in full in S1 Table. Most relevant are the single point mutations encoded by *coq7-5(H153L)* and *coq7-11(W120R)—*both of which severely limit growth on YEPE_3%_ (Fig 1B) without affecting growth on 2% dextrose (YEPD_2%_). We re-integrated select alleles into the original *COQ7* locus of SEY6210 (underlined in Table 1, and designated hereafter by subscript “*i”*), and found that *coq7-11*_*i*_*(W120R)* mutants displayed temperature-sensitive, hypomorphic growth (Fig 1C). During the course of our analyses we also observed that many of the epitope-tagged *COQ7* alleles that have been used in prior studies [22], are also hypomorphic for growth on YEPE_3%_ (Fig 1D). We have assigned allelic designations to some of these genes as well, since they vary in their degree of hypomorphism (Table 1 and S1 Table). Relevant to the current study is *coq7-22(P*_*ADH*_*-COQ7-HA)* which, when carried on a low copy centromeric (CEN) plasmid, conferred only residual growth to *coq7-19(*Δ*coq7)* null mutants cultured on YEPE_3%_ (Fig 1D). We also generated two new epitope-tagged *coq7* alleles, *coq7-9(myc tag)* and *coq7-10(HA tag)*, using the promoter, open reading frame (ORF) and terminator sequences from SEY6210 wild type *COQ7*, and we confirmed that the presence of a C-terminal tag alone results in hypomorphic growth on YEPE_3%_ (Fig 1E), without disrupting growth on YEPD_2%_. Finally, we mapped all newly identified allelic mutations onto a previously published structural model of Coq7p [21], (Fig 1F), the reliability of which has since been supported by spectroscopic and kinetic analyses [34, 35]. *coq7-5(H153L)* disrupts the strictly conserved His153 residue and replaces it with a leucine. His153 chelates one of two iron atoms required for catalysis in the Coq7p active site. *coq7-11(W120R)* replaces the highly conserved Trp120 residue with positively charged arginine. Trp120 resides in a loop predicted to insert directly into the mitochondrial inner membrane. Trp120 also juxtaposes against the C-terminus and we have previously noted that Coq7p orthologs across multiple species all end abruptly with a bulky hydrophobic amino acid (F, I, or L) [21]. Presumably *coq-7-11(W120R)*, as well as *coq7-9(myc tag)* and *coq7-10(HA tag)*, disrupt this juxtaposition.

**Table 1:** *coq7* Allelic Series^†^.

Allele	Key Mutations*	Other Mutations***
*COQ7 (wild type)*	-	
*coq7-1*	G65D	
*coq7-2*	Q48R^**^	
*coq7-3*	L198P	Q48R
*coq7-4*	R159⦸	Q48R, (I222V)
*coq7-5*	H153L	Q48R
*coq7-6*	F15L, V58A	Q48R
*coq7-7*	Q42R, R57H	Q48R
*coq7-8*	V55A, V111D	Q48R
*coq7-9*	C-terminal Myc tag	
*coq7-10*	C-terminal HA tag	
*coq7-11*	W120R	Q48R
*coq7-12*	W120R	Q48R, G65G (silent)
*coq7-13*	T32S, S182P, L195P	Q48R
*coq7-14*	V55D	Q48R
*coq7-15*	S45P, P113S	Q48R
*coq7-16*	V5A, R224⦸	Q48R
*coq7-17*	D59V, K174E	Q48R
*coq7-18*	Q189⦸	Q48R
*coq7-19*	*coq5Δ*::*GFP; HIS3*	
*coq7-20*	*coq7Δ*::*KanMX*_*2*_	
*coq7-21*	E231⦸	I91M, (C-terminal HA tag)
*coq7-22*	*P*_*ADH1*_*-COQ7-HA*	C-terminal HA tag
*coq7-23*	*P*_*ADH1*_*-COQ7*	

^†^ *coq7-1*, *coq7-19* and *coq7-20* are inviable on YEPE_3%._ *coq7-3* to *coq7-18*, *coq7-22* and *coq7-23* are hypomorphic for growth on YEPE_3%_, while *coq7-21* is a hypermorph. Refer to **Fig 1E** for the quantification of allele severity.

^*^ Termination codon indicated by '⦸'. Mutant alleles that were moved back into the *COQ7* locus of SEY62010 are underlined.

** Q48R mutants grow indistinguishably from wild type (from 15–35°C).

*** Mutations in brackets follow premature stop codon. See S1 Table for other silent mutations.

### Identification of *coq7* suppressor genes

**Fig 2:**
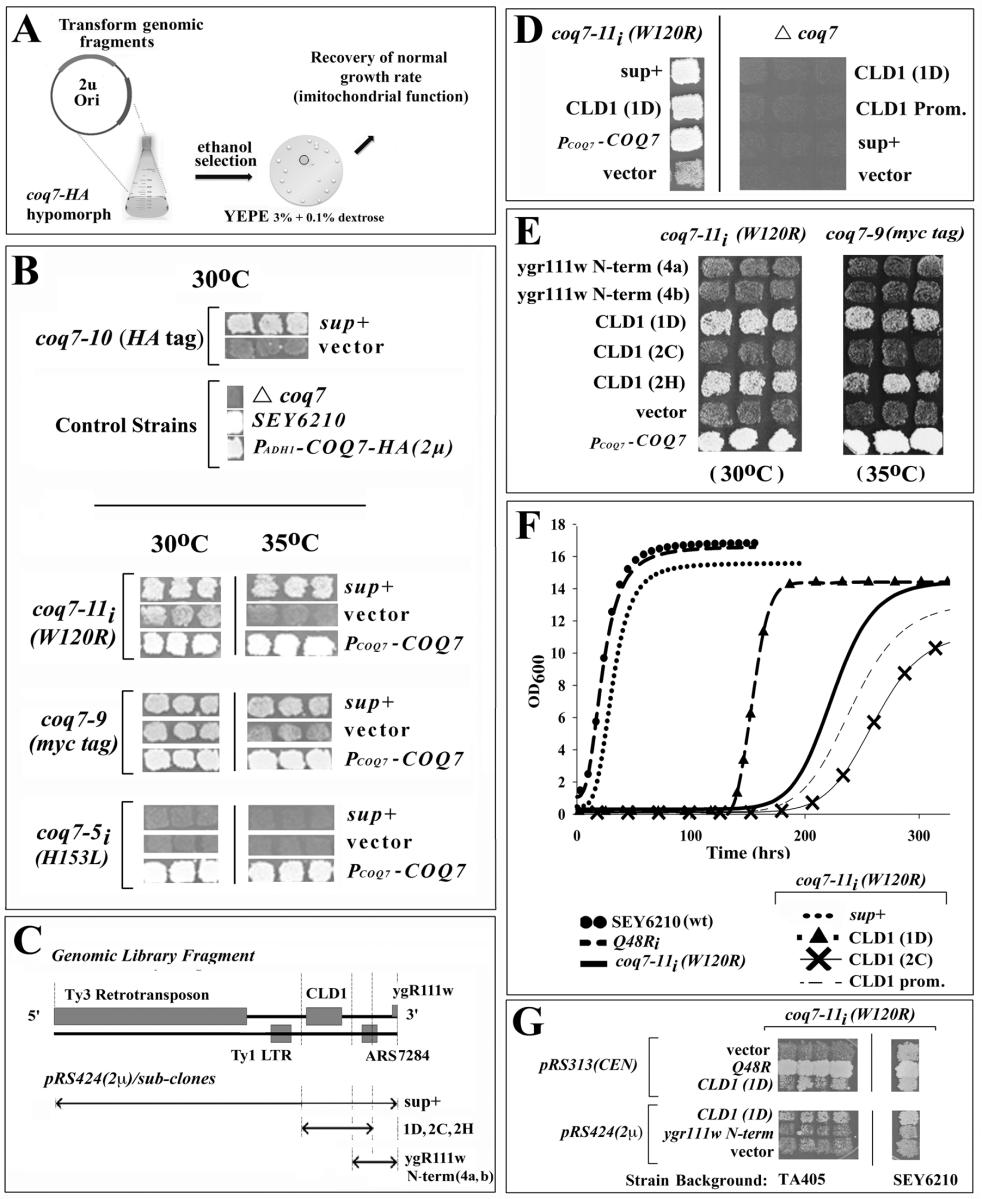
*CLD1* is an Allele-specific Suppressor of Hypomorphic *COQ7*. **(A)** Schematic of genomic library screening protocol: *coq7-19(Δcoq7)* yeast carrying the hypomorphic *coq7-22(P*_*ADH1*_*-COQ7-HA)* allele on a low copy number (CEN) plasmid were transformed with a library of genomic fragments cloned into high-copy plasmid pRS424 (2μ origin of replication). Suppressors of the ‘hypomorphic growth on 3% ethanol’ phenotype of the parent strain were then isolated. **(B)** One library suppressor clone *(sup+)* isolated conferred allele-specific suppression to *coq7-9 (P*_*COQ7*_*-COQ7-Myc (CEN))*, *coq7-10 (P*_*COQ7*_*-COQ7-HA (CEN))*, *coq7-11*_*i*_*(W120R)*, but not *coq7-5*_*i*_*(H183L)*; indicating suppression did not depend upon the HA epitope tag, the *ADH1* promoter, or simply alter plasmid copy number of hypomorphic *P*_*ADH1*_*-COQ7-HA(CEN)*. Both the *coq7-5* and *coq7-11* alleles were re-integrated (subscript *‘i’)* into the wild type *COQ7* locus. Growth enhancement was more pronounced at 35°C for *coq7-10* and *coq7-11*. [vector: pRS424 (2μ)] **(C)** Chromosomal map showing genetic landscape of library suppressor clone *(sup+)* and the fragment boundaries used for various plasmid constructs. 1D, 2C, 2G, 4a and 4b indicate independent PCR amplicons. **(D)** Growth suppression requires residual *COQ7* activity to mediate enhanced growth on 3% ethanol (30°C). Strain genotype (*top)* and transformed plasmid identity (see panel **C**) are indicated. (*Prom*.—promoter). **(E)** The *CLD1* locus is sufficient to confer growth enhancement on 3% ethanol to *coq7-11* (30°C, *left panel*) and *coq7-10* (35°C, *right panel*). Strain genotype (*top)* and transformed plasmid identity (refer to panel **C**) are indicated. **(F)** Quantification of *coq7-11*_*i*_*(W120R)* growth inhibition in 3% ethanol (30°C) and its suppression by overexpression of *CLD1*. Neither the *CLD1* promoter nor the *cld1 loss-of-function* 2C amplicon are able to suppress the slow growth phenotype of *coq7-11*_*i*_*(W120R)*. (wt, wild type). Part of the promoter of *CLD1* was removed when constructing the 1D amplicon to avoid incorporation of the Ty1 LTR into the clone and we presume this is the reason we did not observe the same degree of suppression as the original library clone (sup+). Disruption of just CLD1 within the sup+ clone background did not confer any suppression (see later, main text). **(G)** The ability of *CLD1* to suppress hypomorphic *coq7-11i (W120R)* is independent of strain background. TA405 [*COQ7*/*coq7-11*_*i*_*(W120R)*] heterozygous diploids were sporulated and four independent haploid *coq7-11*_*i*_*(W120R)* isolates retained (*left*). Plasmid DNA (both CEN and 2μ) containing various *CLD1* derivatives were transformed into TA405[*coq7-11*_*i*_*(W120R*)] or control SEY6210[*coq7-11i (W120R)*] yeast *(right)* and then growth enhancement on 3% ethanol quantified at 30°C after 8 days.

To identify new regulators of Coq7p function, we undertook a high copy suppressor screen using the severely hypomorphic *coq7-22(P*_*ADH1*_*-COQ7-HA)* allele. Mutant yeast were transformed with a library of SEY6210 genomic fragments carried on a high copy number plasmid. Growth suppressors were isolated following selection on YEPE_3% + 0.1% DEX._ (Fig 2A). Using this approach we recovered multiple genomic fragments containing a wild type copy of *COQ7—*validating our screening strategy. In addition, a single library suppressor clone *(sup+)* was identified that, when isolated and transformed into other *coq7* mutant backgrounds, conferred suppression to *coq7-9[P*_*COQ7*_*-COQ7-Myc (CEN)]*, *coq7-10[P*_*COQ7*_*-COQ7-HA(CEN)]* and *coq7-11*_*i*_*(W120R)*, but not *coq7-5*_*i*_*(H183L)* (Fig 2B). These findings confirm that suppression by this clone did not depend upon the HA epitope tag, the presence of a Q48R point mutation, or the *ADH1* promoter, and was not simply due to alterations in the copy number of the plasmid upon which hypomorphic *P*_*ADH1*_*-COQ7-HA(CEN)* resided. Intriguingly, this genomic suppressor restored activity to Coq7p in an allele-specific manner–specifically, only *coq7* mutations predicted to affect the membrane association of Coq7p were rescued (Fig 1F).

### Cld1p is a novel modulator of Coq7p function

Analysis of the genomic suppressor fragment revealed that overexpression of CardioLipin-specific Deacylase 1 (*CLD1)*, a gene encoding a phospholipase A_2_ required for cardiolipin-specific acyl remodeling [36–38], was responsible for growth suppression in *coq7-9[P*_*COQ7*_*-COQ7-Myc (CEN)]*, *coq7-11*_*i*_*(W120R)*, *coq7-10[P*_*COQ7*_*-COQ7-HA(CEN)]* and *coq7-22(P*_*ADH1*_*-COQ7-HA)* mutants (Fig 2D–2G, and see later). Consistent with our earlier observation that the suppressing genomic fragment was unable to rescue growth of the severe, *coq7-5*_*i*_*(H183L)* catalytic-site mutant on YEPE_3% + 0.1% DEX._, *CLD1* overexpression was likewise unable to restore growth to *coq7-19(*Δ*coq7)* null mutants on this same media (Fig 2D, *right panel*). These data imply that *CLD1* does not simply substitute for Coq7p activity, consistent with the different enzyme activities of the two proteins.

### *CLD1* suppression occurs independent of the *pet56* mutation

Several yeast laboratory strains contain the *his3-*Δ*200* mutation, including SEY6210—the parental background for our studies. The *his3-*Δ*200* allele contains a 1036 base pair deletion that removes the entire *HIS3* coding region and part of the neighboring promoter region of the *PET56* gene [39]. As a consequence, *PET56* expression is decreased by ~80% [40]. *PET56* is responsible for formation of 2’-O-methylguanosine at position G2251 in the mitochondrial large ribosomal RNA (21S rRNA). This nucleotide sits in the peptidyl transferase center of the mitochondrial ribosome and it has been previously reported that *his3-Δ200* mutants are respiration-deficient when grown at 18°C, and sluggish at 30°C [39]. TA405 yeast contain the *his3-11*,*15* loss-of-function allele which contains two single base pair deletions and does not affect PET56 expression. We exchanged the *COQ7* locus in TA405 with the *coq7-11(W120R)* mutant allele and observed severely retarded growth on YEPE_3%_ which was suppressible by *CLD1* overexpression (Fig 2G). These findings show that *CLD1* is a *bona fide* suppressor of hypomorphic *coq7* mutants.

### Cld1p activity is required for *coq7* suppression

**Fig 3:**
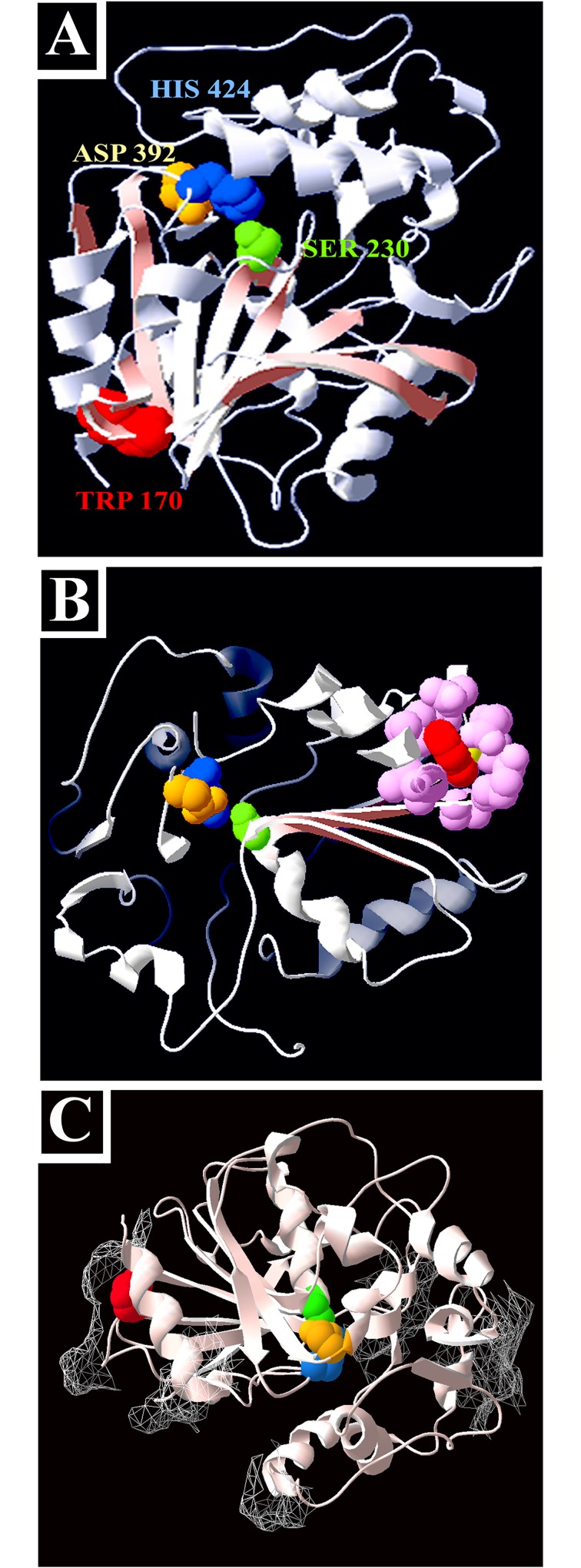
Cld1p contains an α/β hydrolase fold essential for *coq7* suppression. **(A-C)** Homology model of Cld1p, built using PDB structure 2CJP.A (*Solanum tuberosum* epoxide hydrolase 1). Highlighted features include–(A-C) the catalytic triad [Ser230 (*green*), Asp392 (*yellow*) His424 (*blue*)]; the W170R point mutation encoded by the non-suppressing *cld1* ‘2C amplicon’ (*red);* (in B only) structural residues of the α/β hydrolase fold immediately surrounding Trp170 (*pink);* and (in C only) surface-exposed hydrophobic patches (mesh).

While examining the role of *CLD1* in the recovery of *coq7* function, we isolated a *cld1* mutant that was completely blocked in its ability to rescue the growth of any hypomorphic *coq7* allele (‘clone 2C’ in Fig 2E and 2F). Sequence analysis revealed a PCR-induced point mutation that converted Trp170 to arginine (W170R). It has been previously reported that Cld1p contains an α/β hydrolase fold [38]. As for other hydrolases belonging to this family, the fold acts as a scaffold to correctly position a catalytic triad of residues situated on the end of three distal loops. The α/β hydrolase family has evolved to accommodate a wide range of substrate specificities because only the triad and shape of the fold are essential to catalysis [41]. We generated a model of Cld1p based on the crystal structure of epoxide hydrolase 1 from *Solanum tuberosum* (Fig 3A–3C). We identified the active site residues as Ser230, Asp392 and His424. This model is in good agreement with the model constructed by Baile and colleagues [37] using the α/β hydrolase domain of CumD. These authors experimentally verified that Ser230, Asp392 and His424 were each essential for Cld1p activity. Surprisingly, Trp170 sits distal from the catalytic triad (Fig 3A), suggesting W170R must disrupt Cld1p function indirectly—possibly through destabilization of the α/β hydrolase fold (Fig 3B), or by disrupting membrane binding (Fig 3C). In regards to the latter, Cld1p is a membrane protein that faces the mitochondrial matrix [37]. Our model shows that Cld1p is surrounded by a skirt of surface-exposed hydrophobic patches, consistent with its function as a membrane lipid-modifying enzyme, and W170R sits directly below one of these regions (Fig 3C).

### Overexpressing Cld1p re-models acyl composition of MLCL in *coq7-11*_*i*_*(W120R)* mutants

**Fig 4:**
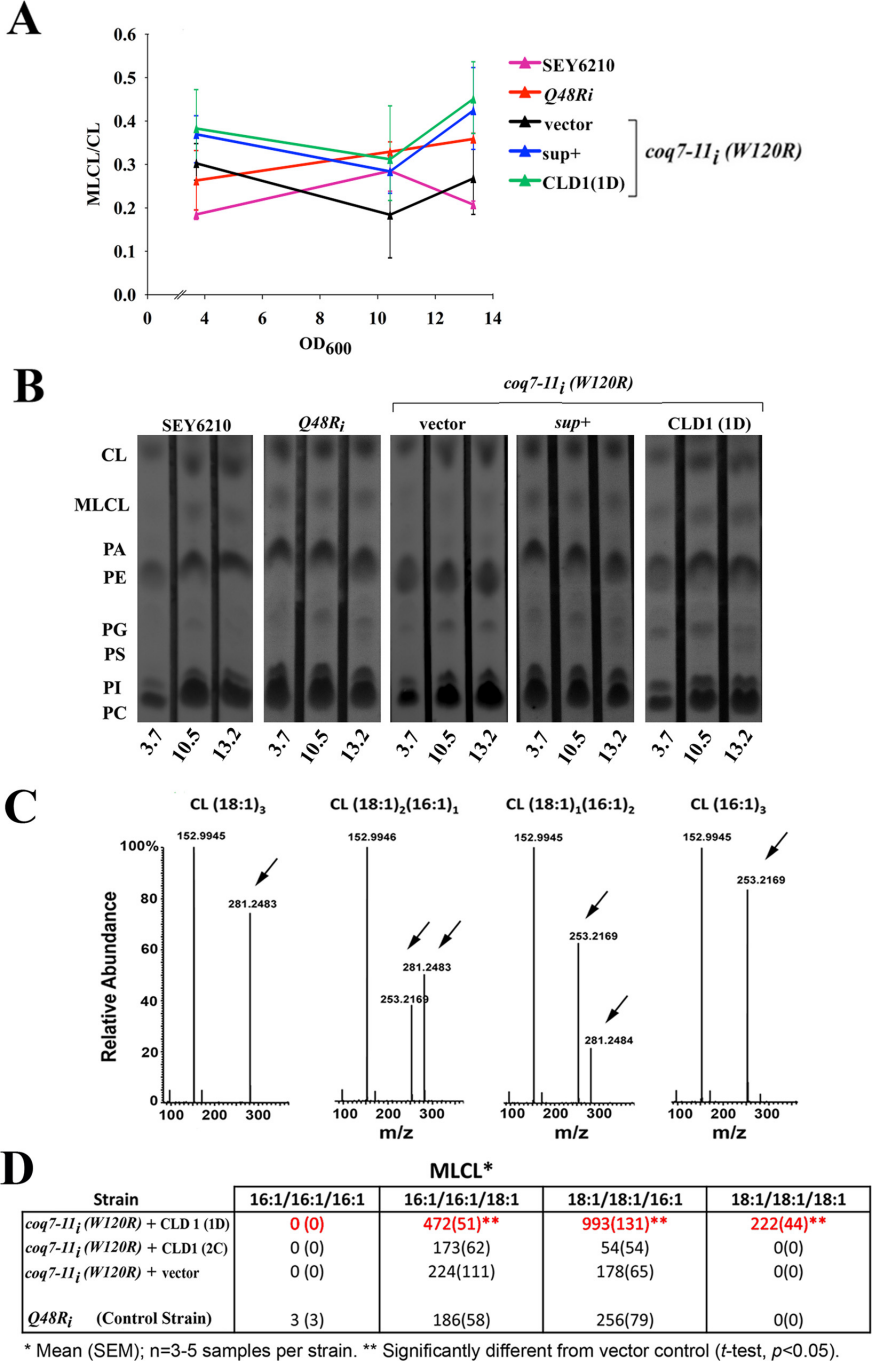
CLD1 overexpression alters whole-cell monolysocardiolipin content of *coq7* hypomorphs. **(A, B)** Whole-cell lipid extracts were prepared from the indicated yeast strains following growth at 25°C in liquid YEPE_3%_ to the indicated optical density (OD_600_). Lipids were analyzed by thin layer chromatography (TLC). In (A), average MLCL/CL ratios (+/- range) are plotted for duplicate independent experiments, with triplicate technical replicates assayed at each OD_600,_ in each experiment. Multiple regression analysis (S2 Table) revealed a significant (*p<0*.*007*) increase in the MLCL/CL ratio in *coq7-11*_*i*_*(W120R)* yeast following overexpression of Cld1p (1D and sup+). In (B) representative, raw TLC data is shown. CL, cardiolipin; MLCL, monolysocardiolipin; PA, phosphatidic acid; PE, phosphatidylethanolamine; *PG*, phosphatidylglycerol; *PS*, phosphatidylserine; *PI*, phosphatidylinositol; *PC*, phosphatidylcholine. **(C)** MLCL derivatives were extracted from yeast cultured at 25°C to mid-log phase (OD_600_ ~7), then analyzed by HPLC-ESI-MS/MS. *m/z* for [M-H]^-^ of CL(16:1/16:1/16:1), CL(16:1/16:1/18:1), CL(18:1/18:1/16:1) and CL(18:1/18:1/18:1) used for quantification are m/z 1107.6884, 1135.7196, 1163.7510 and 1191.7823, respectively. *Arrows* indicate C16:1 and C18:1 peaks used for MS1 identification. **(D)** Absolute quantitation of MLCL species in mid-log phase of the indicated yeast strains (pmole per 35ml culture volume, OD_600_ 7.0). Data is presented as mean (+/- SEM), n = 4–5 independent replicates per strain. Significance testing was undertaken using Student’s *t-test p<0*.*05)*. Overexpression of wild type CLD1 (amplicon 1D) in the *coq7-11i(W120R)* background leads to a shift in C18:1 enriched MLCL species. This shift was not observed when the non-suppressing *cld1(W170R)* point mutant (encoded by amplicon 2C) was overexpressed, suggesting this allele either directly or indirectly results in an enzymatic loss-of-function protein.

In yeast, Cld1p deacylates cardiolipin (CL) to generate monolysocardiolipin (MLCL) and this enzyme shows a preference for removing palmitic acid (C16:0) [38]. Once formed, MLCL can be re-acylated with longer (C18) unsaturated fatty acids by the Tafazzin ortholog, Taz1p [36]. When Taz1p is removed from cells MLCL accumulates to detectable levels, suggesting Cld1p is constitutively active [42]. Cld1p and Taz1p therefore work together to dynamically control cardiolipin remodeling in the mitochondria [38]. It has been previously reported that wild type cells overexpressing Cld1p display no detectable alteration in their relative ratio of MLCL to CL [38], suggesting Taz1p, and not Cld1p, is rate limiting for CL production in yeast. In contrast, when Cld1p was overexpressed in *coq7-11*_*i*_*(W120R)* cells we saw a significant increase in the relative ratio of MLCL to CL in whole-cell lipid extracts (Fig 4A and 4B, Multiple Regression Analysis, p<0.007). Cardiolipin contains four acyl chains, the composition of which influences CL functionality [43]. To examine the enzymatic activity of Cld1p further, we investigated how overexpressing Cld1p changed the acyl composition of CL in *coq7-11*_*i*_*(W120R)* cells. We used HPLC-ESI-MS/MS and focused specifically on the product of the Cld1p reaction, MLCL. Four dominant MLCL species have been previously described in *S*. *cerevisiae* [44]. We observed a marked shift in the acyl composition of the MLCL pool in *coq7-11*_*i*_*(W120R)* cells overexpressing *CLD1* relative to control lines (Fig 4C and 4D). Notably, MLCL species containing C18:1 became significantly more abundant (*t-test*, p < 0.05). Interestingly, the appearance of MLCL(18:1)_3_ in these cells suggests Cld1p may have both phospholipase A_2_ and A_1_ activity, and that this enzyme might be more appropriately described as a phospholipase B [45]. In contrast, *coq7-11*_*i*_*(W120R)* cells overexpressing the *cld1(W170R*) mutation (‘clone 2C’) showed that the protein encoded by this allele was effectively enzymatically dead, which again emphasized the necessity of functional Cld1p for phenotypic rescue. Overall, our findings showing direct changes in the abundance of the MLCL product of the Cld1p reaction are in agreement with an earlier study that showed removal of *CLD1* decreased the abundance of CL species containing unsaturated acyl chains [36].

### The Q_6_/DMQ_6_ ratio is normalized in *coq7* mutants following *CLD1* overexpression

**Fig 5:**
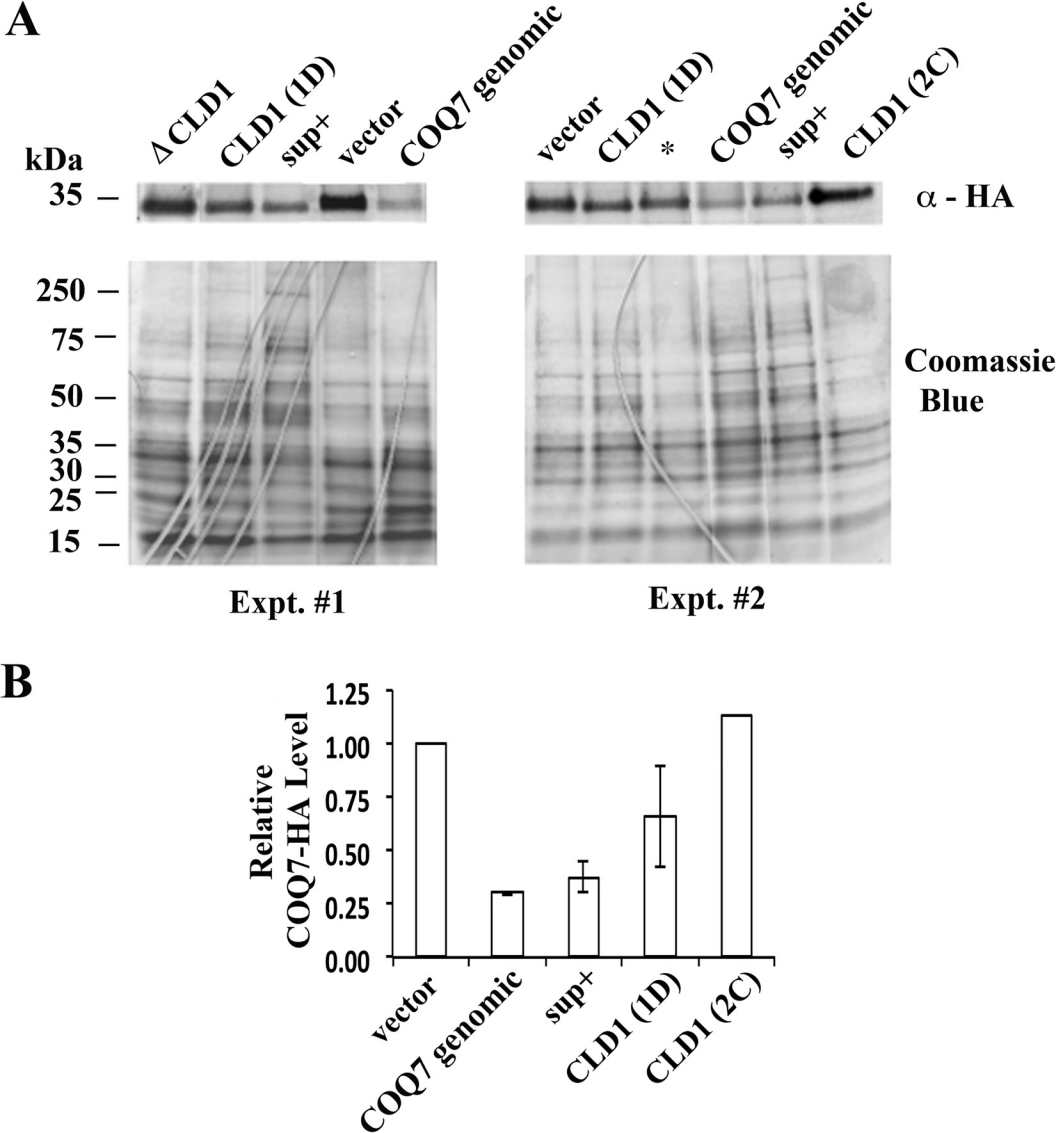
Effect of Cld1p over-expression on the protein level of Coq7p. (**A)** A *coq7* null mutant [*coq7-19(coq7Δ*::*GFP; HIS3)*] containing the hypomorphic *coq7-22(P*_*ADH1*_*-COQ7-HA)* allele on a centromeric (CEN) plasmid, was transformed with the indicated genomic fragment contained on a high copy number (2-micron, 2μ) plasmid (pRS424). Cells were cultured at 25°C in YEPE_3%_ until reaching stationary phase, then whole cell lysates collected and analyzed for Coq7p-HA expression by western analysis. Raw data from two independent experiments are shown. **(B)** Relevant lanes in (A) are quantified in panel B. Average and range are shown. In (A), ΔCLD1 is the original CLD1-containing genomic suppressor (*sup+*) library clone with the CLD1 open reading frame deleted. The asterisk in the *right panel* of (A) is a second library suppressor clone that was identified but which is not discussed further in this study.

**Fig 6:**
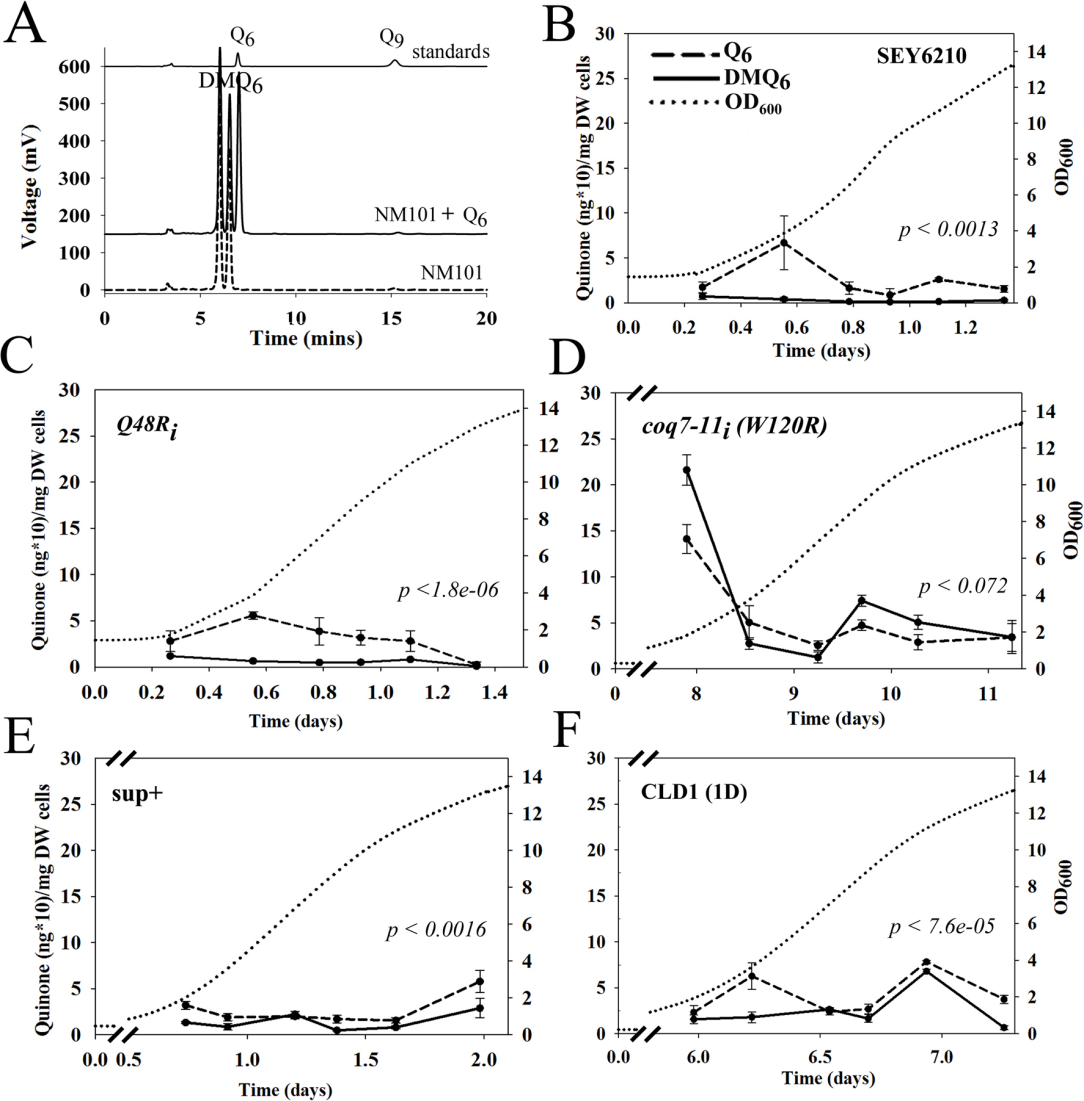
Effect of CLD1 Overexpression on Cellular Quinone Levels. **(A)** Quantitation of Q_6_ and DMQ_6_ levels in yeast whole-cell extracts using UV-HPLC. Three traces are offset on the vertical axis. *Bottom trace*: extract from loss-of-function NM101[*coq7-1(G65D)*] mutant containing DMQ_6_ (*right* peak)*; middle trace*: extract from NM101 co-injected with Q_6_ standard; *top trace*: purified Q_6_ and Q_9_ standards. Q_9_ was added as an internal standard for all yeast quinone extractions. Peaks were detected using λ_275nm_. **(B-F)** UV-HPLC quantitation of Q_6_ (*dashed line*) and DMQ_6_ (*solid line*) in the indicated strains following growth in liquid YEPE_3%_. Quinone content is shown relative to culture density (OD_600,_ *dotted line*). Broken axes highlight the temporal difference in growth rate between strains (abscissa). (E) and (F) represent *coq7-11*_*i*_*(W120R)* yeast containing the indicated construct (see Fig 2C for details). Each data point is the average of three independent biological replicates and represents total (reduced + oxidized) Q_6_ and DMQ_6_. Error bars indicate standard deviation (See S3 Table for statistical analyses). *p-values* indicate strength of significance for whether Q_6_ and DMQ_6_ profiles differ (significance threshold after Bonferroni correction = 0.0038)

We next sought to determine how *CLD1* overexpression suppressed the hypomorphic growth of mutant *coq7* yeast. We observed by western analysis that hypomorphic *coq7-22(P*_*ADH1*_*-COQ7-HA)* mutants overexpressing a genomic fragment containing wild type *COQ7* produced less *COQ7-HA* protein. Overexpression of *CLD1* induced the same effect, while overexpression of mutant *cld1(W170R)* did not (Fig 5). Q_6_ is known to feedback and regulate the stability of its biosynthetic complex [14], of which Coq7p is a component, so our findings suggest that *CLD1* overexpression may be leading to direct recovery of Coq7p activity. We therefore quantified quinone amounts in *CLD1* overexpressing cells during growth on YEPE_3% + 0.1% DEX._ (Fig 6A). We analyzed several strains and observed that SEY6210 (wild type) and *coq7-2*_*i*_*(Q48R)* cells both showed a burst of Q_6_ production shortly after entering log phase growth on ethanol (Fig 6B and 6C). We observed only small amounts of DMQ_6_ accumulate in both strains under these growth conditions. *coq7-11*_*i*_*(W120R)* cells exhibited a similar trend in Q_6_ production at the onset of log phase growth, but it was much more exaggerated in magnitude. Notably, *coq7-11*_*i*_*(W120R)* cells took 8 days to reach early log phase (25 times longer than wild type), and moreover, in contrast to the control lines, by this phase of growth DMQ_6_ levels were also overtly elevated in this strain (Fig 6D). Introduction of the original genomic DNA library suppressor clone (sup^+^) into *coq7-11*_*i*_*(W120R)* cells resulted in reversion of both the Q6 and growth phenotype toward that of the control *coq7-2*_*i*_*(Q48R)* line (Fig 6E and S3 Table). Introduction of just the CLD1 open reading frame showed partial rescue of both phenotypes (Fig 6F), and lack of full suppression was presumably because the flanking DNA control regions were not present in their entirety in this clone (Fig 2C and [37]). Together, these data strongly suggest that *CLD1* overexpression overrides the predicted membrane interacting defects of hypomorphic Coq7p proteins to allow them access to their DMQ_6_ lipid substrate.

### *CLD1* overexpression lengthens the replicative lifespan of *coq7-11*_*i*_*(W120R)* mutants

**Fig 7:**
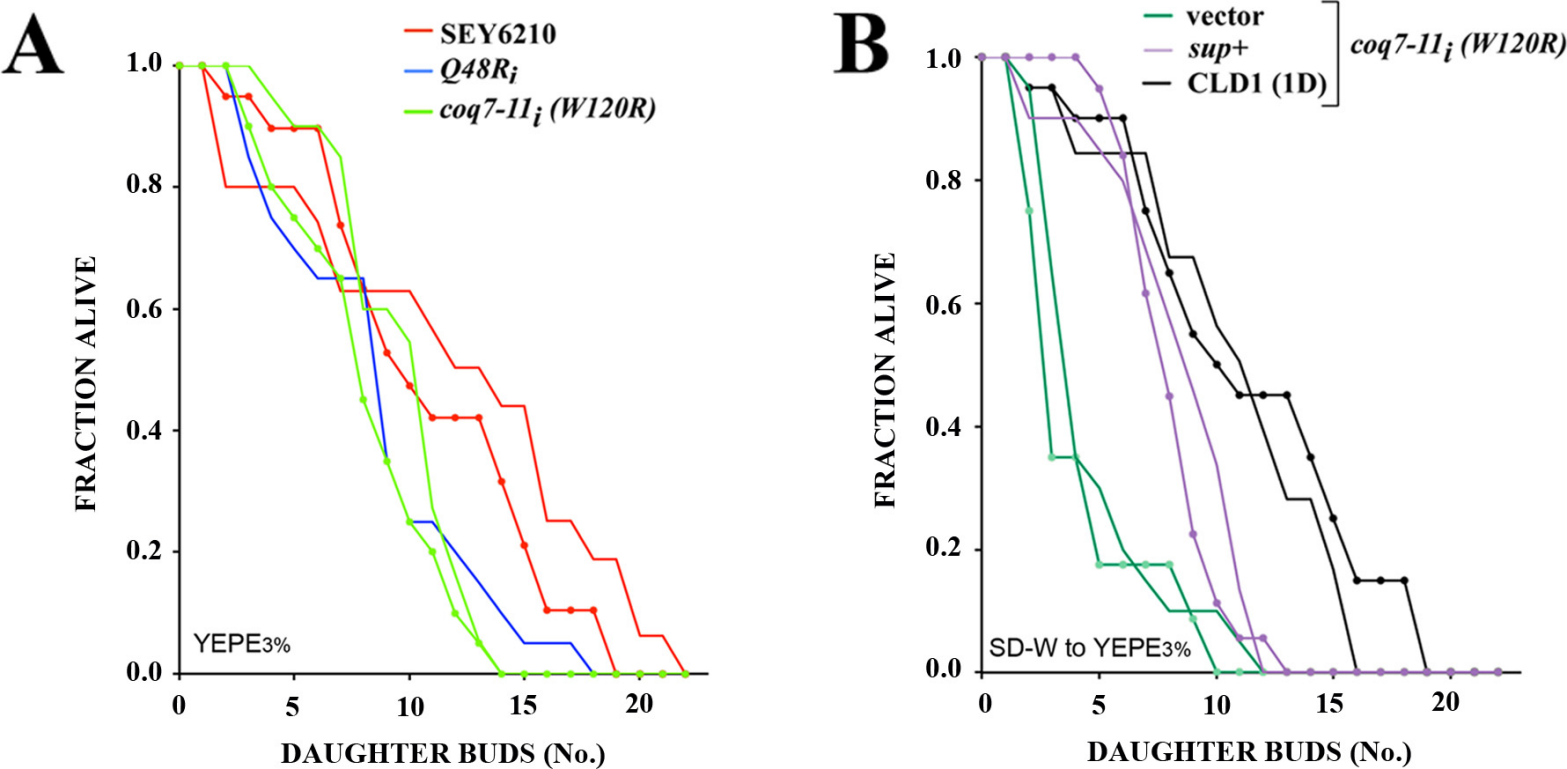
CLD1 overexpression lengthens the replicative lifespan of *coq7* hypomorphs. **(A, B)** Replicative lifespan analyses of the indicated yeast strains cultured at 25°C on YEPE_3%_. Duplicate lifespan experiments for each line are provided (n = 20 virgin mother cells per strain per experiment). The following strain comparisons were significantly different (*p<0*.*05*) after pooling data across replicate experiments [mean replicative lifespan, *p-value* (log rank test)]. In panel (A), *Q48Ri* vs. SEY6210 (6.1 vs. 9.2, p<0.0009), *coq7-11i(W120R)* vs. SEY6210 (6.8 vs. 9.2, p<0.0008). In panel (B), vector (pRS424) vs. *sup+* (2.4 vs. 6.3, *p~0*), vector vs. CLD1 (1D) (2.4 vs. 9.1, *p~0*), and CLD1 (1D) vs. *sup+* (p < .002). *coq7-11i(W120R)* derivatives were cultured on synthetic selective media prior to transferal to YEPE_3%_. All other strains were maintained continuously on YEPE_3%._

Mutations that reduce or remove *clk-1/coq7* function in *C*. *elegans* result in life extension [25]. It has not been possible to determine the effect of complete *coq7* removal on yeast cell survival because Coq7p is essential for growth on YEPE_3%_. With the identification of hypomorphic *coq7* alleles we can now examine the effect of reduced *coq7* function on yeast survival. We measured replicative lifespan, which monitors the ability of individual ‘mother’ cells to generate buds, and is generally considered the functional equivalent of lifespan studies in higher organisms [46]. *coq7-11*_*i*_*(W120R)* mutants showed no significant alteration in replicative lifespan when compared with *coq7-2*_*i*_*(Q48R)* cells, their relevant control (Fig 7A). Interestingly, *coq7-2*_*i*_*(Q48R)* cells, generated significantly fewer buds than SEY6210 yeast cells (Fig 7A and S4 Table). This is unexpected since, by all prior measures these two strains behaved indistinguishably (Figs 1E, 2F and 6). We next examined the effect of *CLD1* overexpression on the replicative capacity of *coq7-11*_*i*_*(W120R)* cells. Under the specific assay conditions we employed (where cells were maintained on auxotrophic, plasmid-selection media immediately prior to analysis on 3% ethanol), the replicative lifespan of *coq7-11*_*i*_*(W120R)* cells was extended between 2- and 3-fold when overexpressing CLD1 (Fig 7B). These findings underscore the major finding of this work–that some forms of ubiquinone insufficiency can be fully reversed by cardiolipin remodeling in yeast.

## Discussion

In this study, we sought to identify genes that, when overexpressed, could suppress the ubiquinone insufficiency caused by hypomorphic disruption of the Q biosynthetic enzyme Coq7p. Using a newly-generated panel of *coq7* mutants in the yeast *S*. *cerevisiae*, we discovered that overexpression of *CLD1*, encoding a cardiolipin-specific phospholipase A_2_ [38], was able to fully rescue the slow growing phenotype of several different *coq7* mutants. We provide evidence that rescue by *CLD1* overexpression is dependent on the enzymatic function of Cld1p, and requires residual Coq7 activity. We have found through structural modeling that the mutant Coq7p proteins that were suppressed by Cld1p all had mutations positioned in, or adjacent to, a predicted membrane-binding region. We therefore cautiously speculate that in the absence of Cld1p overexpression, these mutant Coq7p proteins were unable to insert into the mitochondrial inner membrane efficiently or, if they were, they were unable to adopt the correct depth or orientation in the membrane to permit DMQ_6_ access to the otherwise intact di-iron active site [21, 34, 35]. Consistent with this interpretation, Cld1p overexpression was incapable of suppressing the hypomorphic *coq7-5(H153L)* mutation which encodes a H153L lesion that directly disrupts the Coq7p catalytic site.

The most important finding of our present study is the identification of a functionally significant interaction between a cardiolipin remodeling enzyme and an enzyme of the Q biosynthetic machinery. We showed that enzymatically active Cld1p, when overexpressed, could normalize Q_6_ and DMQ_6_ levels in mutant *coq7-11i(W120R)* yeast. The extent and kinetics of this suppression is apparent in Fig 6: In control yeast grown in non-fermentable media (Fig 6B and 6C), Q_6_ levels reproducibly exhibit a spike upon entry into log-phase growth (refer to OD_600_ axis). A second, minor peak is also observed in SEY6210 cells just before cells reach stationary phase. This latter peak may reflect activation of a stress response as nutrients become limiting, or it may reflect a drop in Q_6_ levels below some critical threshold, in which case Q_6_ synthesis presumably becomes reactivated. In any case, in contrast to Q_6_ levels, DMQ_6_ is maintained at almost undetectable levels during the entire growth phase. The situation for hypomorphic *coq7-11i(W120R)* mutants is, however, very different. We recorded a remarkable elevation of both Q_6_ and DMQ_6_ in these cells as they entered log-phase growth. At first this appears counterintuitive for an allele that is obviously hypomorphic for growth. However, it is clear from Fig 6D that DMQ_6_ levels in this mutant almost always remain higher than those of Q_6_. Due to technical restrictions we were unable to collect sufficient cells to monitor earlier growth points than those shown, but we speculate that hypomorphic *coq7-11i(W120R)* mutants first accumulate massive amounts of DMQ_6_. At first, small amounts of Q_6_ would be synthesized by the mutant enzyme, however, once Q_6_ levels reach a critical threshold, cells enter log-phase and a much more rapid expansion of the mitochondrial network could then ensue. We speculate that either additional Coq7p is then synthesized, which would accelerate the conversion of the remaining DMQ_6_ to Q_6_, as observed, or perhaps Cld1p is naturally activated as part of the mitochondrial expansion, given it is a cardiolipin modifying enzyme and cardiolipin is the most abundant lipid in the mitochondria. Regardless, in the context of *coq7-11i(W120R)* mutants, both options would result in growth enhancement. In support of this idea we note there is a second spike in the quinone profile of *coq7-11i(W120R)* mutants, toward the beginning of stationary phase. Here, DMQ_6_ and Q_6_ are present in much greater amounts relative to the equivalent growth phase of controls cells; and DMQ_6_ levels are almost twice those of Q_6_. Again, this is consistent with a delay in DMQ_6_ processing. The original library clone containing CLD1 in its genomic context conveyed near perfect suppression of the *coq7-11i(W120R)* mutant in regard to its quinone profile (Fig 6E). Overexpression of the Cld1p open reading frame was also effective at suppressing the slow growth defect of *coq7-11i(W120R)* mutants, though with delayed temporal efficacy relative to the genomic clone (Fig 6F), likely because part of the CLD1 promoter region was removed (Fig 2 and [37]). Comparison of quinone levels between strains at various growth stages (compare OD_600_ axes) clearly reveals the extent to which CLD1 overexpression is able to normalize DMQ_6_ and Q_6_ levels in *coq7-11i(W120R)* mutants. In summary, while natural elevation of Cld1p during growth could provide a simple explanation for why CLD1 overexpression was identified in our screen, less easy to explain is how Cld1p acts to suppress hypermorphic Coq7p in order to recover enzyme functionality.

Our finding that overexpression of Cld1p did not change the MLCL/CL ratio (Fig 4A and 4B), but instead increased the average length and degree of unsaturation of the acyl chains in its enzymatic product, monolysocardiolipin (Fig 4C and 4D), suggests that a shift in the inner membrane lipid composition likely plays an important role in the rescue of hypomorphic *coq7* mutants. To date, there has been no report of Coq7p directly binding either cardiolipin or MLCL. In *S*. *cerevisiae*, cardiolipin species fall into five prevalent sub-types: CL(16:1)_4_; CL(16:1)_3_(18:1)_1_; CL(18:1)_2_(16:1)_2_; CL(18:1)_3_(16:1)_1_ and CL (18:1)_4_ [44]. Even so, this amounts to 13 possible CL isomers, more when minor acyl species are included, and even more when ^13^C isotopomers are included; understandably the precise molecular profile of CL in yeast remains poorly uncharacterized. It has been reported, however, that by late log phase the relative abundance of C16:0, C16:1, C18:0 and C18:1 acyl groups is 30%, 30%, 5% and 35%, respectively [38]. Our MS data clearly showed that *CLD1* overexpression increased the abundance of C18:1 in MLCL. We do not know if alterations to MLCL *per se*, or to CL were required for hypomorphic Coq7p functional recovery, but we believe the most simple interpretation of our data is that changes in the mitochondrial lipid environment allowed previously hypomorphic variants of Coq7p to assume full catalytic functionality. Phospholipids are known to provide a conducive environment for the activity of many membrane proteins by dictating their folding and assembly [47, 48]. The activity of many integral proteins is also dependent upon lipid bilayer thickness, which in turn is dependent upon acyl chain length [49, 50]. Lipid-specific binding sites on integral membrane proteins are also known [47, 51], and in this regard computational studies indicate that the side chains of hydrophobic residues such as isoleucine, leucine, valine and phenylalanine often interact with the acyl chains of phospholipids, while the more polar residues interact with lipid polar head groups and their glycerol backbones [47]. We found that the mutation in *coq7-11i(W120R)* replaces the highly hydrophobic tryptophan 120 residue with arginine. Since tryptophan does not generally tolerate substitution because of its structural uniqueness, in the absence of cardiolipin data, we cautiously speculate that arginine changed the hydrophobic surface of Coq7p enough to impair its interaction with the acyl chains of cardiolipin, and that Cld1p caused a compensatory change in the cardiolipin acyl profile that led to recovery of Coq7p activity in the mutant. Finally, it is possible that MLCL/CL substitution with C18:1 simply affected the thermal stability of mutant Coq7p proteins, a previous example of which is found in a site directed mutagenesis study involving interaction between a diacidic molecule of cardiolipin and the purple bacterial reaction center [48].

While alterations to the mitochondrial lipid environment that in turn directly affect Coq7p’s ability to insert into the mitochondrial inner membrane is our favored hypothesis for how CLD1 overexpression functions to suppress hypomorphic Coq7p mutants with predicted membrane-binding defects, alternate explanations do exist. All are less-strongly supported by our data. For example, one possibility is that mutant Coq7p proteins with reduced membrane-binding capacity are toxic to yeast cells and Cld1p overexpression somehow reduces their level. Although we showed that Cld1p overexpression decreased the level of mutant Coq7p protein in *coq7-22(HA-tag)* yeast, ubiquinone levels went up, not down, arguing against this hypothesis. Moreover, *coq7-11(W120R)* mutants, which are suppressible by CLD1 overexpression, exhibited no evidence of toxicity with respect to replicative lifespan when compared to control *coq7-2*_*i*_*(Q48R)* cells, further arguing against this idea. Another alternate explanation is that the shift in the acyl signature of MLCL (and presumably cardiolipin by extension) toward C18:1, led to enhanced mitochondrial electron transport chain activity in hypomorphic *coq7* mutants, and this was the reason they recovered their ability to grow on ethanol. Consistent with this idea, it is well established that mitochondrial respiratory chain complexes require cardiolipin for their structural and functional integrity (reviewed in [43]). Against this hypothesis, however, is our observation that the catalytically-compromised *coq7-5(H153L)* hypomorph was not equally rescued by CLD1 overexpression. Also, we observed a marked recovery in Q production in suppressed cells, again suggesting that the suppressive effect of Cld1p overexpression was specific to the Coq7p protein and not due to supplemental enhancement of ETC activity.

Recently, Busso and colleagues [52] also made use of random PCR mutagenesis in an effort to probe the structure/function of Coq7p. They identified only a single and weakly hypomorphic allele of *coq7*, *(D53G)*, which contrasts with the 78 that we identified in this study, highlighting the strength of our new screening approach. These authors turned to an automated server-generated structural model of Coq7p and, in conjunction with site-directed mutagenesis, tested the function of several amino acid changes in the vicinity of D53G. Using this approach, they discovered a novel *coq7(S114E)* mutant which accumulated DMQ_6_ at the expense of Q_6_, and which was severely hypomorphic for growth on ethanol/glycerol at 30°C, and lethal at 37°C. Overexpression of Coq8p partially rescued the growth defect. When *coq7(S114E)* cells were placed at the non-permissive temperature of 37°C, the stability of Coq3p and Coq4p halved. This was not observed at 30°C, even though these mutants remained severely hypomorphic for growth on ethanol/glycerol. This finding suggests that formation of the 700kDa Q biosynthetic pre-complex was not limiting in *coq7(S114E)* mutants at lower temperatures and that some other function of Coq7p was disrupted. S114E maps to the same region of Coq7p where all our Cld1p-suppressible mutations localize. As mentioned above, this region is predicted to be surface-exposed, hydrophobic, membrane-binding, interfacial with the Coq7p C-terminus, and sit directly underneath the active site where the DMQ_6_ tail extends [21].

Finally, Freyer and colleagues [6] recently reported the first known *COQ7* mutation in a patient with primary Q_10_ deficiency. The Freyer study demonstrated that administration of 2,4-dihydroxybenzoic acid (2,4DHB), a quinone analogue containing the ring modification normally catalyzed by *COQ7*, rescued the biochemical defect in patient-derived fibroblasts. This same compound was shown in an earlier study to essentially cure a mouse model of *Coq7* deficiency [53]. Both studies had their genesis in yeast work using *coq6* mutants and related quinone analogues [54, 55]. Together, these findings highlight the non-linearity of Q biosynthesis and portend what will hopefully be a brighter future for patients with Q_10_ deficiency. These findings also underscore the exciting conclusion from our study, that not only is there potentially another way to treat Q_10_ deficiency in humans, albeit not as elegant as the solution above, but more importantly that we have broadened the field towards identifying alternate mechanisms for potentially reversing other deficiencies that result from alterations in the activity of critical membrane-bound enzymes.

## Materials and Methods

### Strains

Strain information for all lines generated in this study is provided in S1 Table. Stock lines, SEY6210 (*MATα leu2-3*,*112 ura3-52 his3-Δ200 trp1-Δ901 suc2-Δ9 lys2-801; GAL)* and TA405 (*MATα/a his3-11*,*15/ his3-11*,*15 leu2-3*,*112/ leu2-3*,*112 can1/can1*) were maintained on rich media [1% (w/v) yeast extract, 2% peptone, 2% dextrose (YEPD_2%_) + 2% agar]. Strains containing labile plasmids were maintained under constant selection using either 3% ethanol as carbon source (YEPE_3%_), or on synthetic dropout (SD) media supplemented with 2% glucose.

### Generation of *coq7* allelic series

A 1371 base pair (bp) fragment containing the *COQ7* gene was PCR amplified from SEY6210 and cloned into the BamH1 and Sal1 sites of pRS315(CEN) using forward (5’-CGCGGATCCGCTAGATGATGGATCTAAC-3’) and reverse (5’-GGACGCGTCGACGTTCATTATCTTCGTTCGGCATTTCC-3’) primers to form pRS315/*COQ7*. Mutagenic PCR, using Taq DNA polymerase, 40 μM Mn^2+^, 20 rounds of thermal cycling and the same primer pair listed above, was then used to randomly introduce ~1 error/kbp along the entire 1371 bp *COQ7* fragment. The resulting ensemble of PCR products was then directly transformed into strain SHM1 [SEY6210 *Δcat5/coq7-19*::*GFP;HIS3*] [56] along with an equal molar amount of purified pRS315/*COQ7* previously cut with SnaB1 and HindIII. Both restriction sites reside within the *COQ7* locus–approximately 50 bp from the 5’ and 3’ end, respectively. Homologous recombination occurred with high enough frequency to make this a viable alternative to cloning the PCR products manually. This and all subsequent transformations were undertaken using lithium acetate. Transformants containing the recombined plasmid were selected on glucose-containing minimal media lacking leucine (SD-L + 2% glucose) at either 25°C (2300 recombinants) or 30°C (1300 recombinants). Plasmid alone recombined with a frequency of <0.2% of the total number of positives. After 3 days, colonies were doubly replica plated onto YEPE_3% + 0.1% DEX._ and grown at 25°C and 37°C, or 30°C and 37°C, respectively, and hypomorphic or temperature-sensitive *coq7* alleles isolated. Seventy-eight mutants in total were identified. Plasmids were recovered from a sub-collection of these yeast and their entire *COQ7* insert sequenced bi-directionally. Several alleles were re-integrated back into the *coq7*Δ locus of SHM1 (S1 Table). To do this, mutant *coq7* alleles were excised from their pRS315 vector backbone using BamH1 and SalI and cloned by homologous recombination into the SHM1 *coq7*Δ locus. Integrants were selected on YEPE_3%_ at a temperature permissive for growth (S1 Table), and insertion at the *coq7*Δ locus was confirmed by PCR. The HA-epitope-tagged *coq7* alleles in psHA71 and pmHA71 have been described previously [22]. Both alleles are under the expression of the *ADH1* promoter. Sequence analysis revealed both constructs also differ from the *COQ7* sequence present in SEY6210 at position 177 (M to I). This is a naturally-arising Coq7p polymorphism that confers resistance to petite formation [57]. NM101 (*MATa coq7-1 leu2-3*,*112 ura3-52 his3*^*-*^*)*, which contains the previously described *coq7-1(G65D)* allele [15], was the original source of genomic DNA used for PCR generation of psHA71 and pmHA71.

### *COQ7-HA* and *COQ7-myc* construction

Wild type *COQ7* was tagged with a myc- or haemagglutinin- (HA) epitope as follows: Site-directed mutagenesis was used to introduce an EcoR1 site at the C-terminus of *COQ7* in the plasmid pRS315/*COQ7* using the following primer pair (EcoR1-5’): 5’-GGAGTGCCGAAAGAATTCAACCACCAGAAAGTGGC-3’ and (EcoR1-3’): 5’-GCCACTTTCTGGTGGTTGAATTCTTTCGGCACTCC-3’. Next, primer pairs encoding the *myc-* or HA- epitope were annealed, then directly ligated into the newly generated EcoR1 site to form pRS315/*coq7-9* and pRS315/*coq7-10* respectively: (myc5’): 5’-AATTGGGGGGGAGGAGCAGAAGCTGATCTCAGAGGAGGACCTGCATATGTAA-3’, (myc3’): 5’-AATTTTACATATGCAGGTCCTCCTCTGAGATCAGCTTCTGCTCCTCCCCCCC-3’; (HA5’): 5’-AATTGGGGGGTACCCATACGACGTACCAGATTACGCTCATATGTAA-3’, (HA3’): 5’-AATTTTACATATGAGCGTAATCTGGTACGTCGTATGGGTACCCCCC-3’.

### *P*_*ADH1*_*-COQ7-HA (CEN)* suppressor screen

Yeast strain SHM1, containing the *coq7-19 (cat5Δ*::*GFP;HIS3)* null allele, was transformed with psHA71 [22]. This plasmid carries the hypomorphic *coq7-22(P*_*ADH1*_*-COQ7-HA)* allele on a pRS316 (CEN) vector backbone. Alone, *coq7-22(P*_*ADH1*_*-COQ7-HA)* conferred only severely hypomorphic growth on YEPE_3%_ at all tested temperatures (15–37°C). The resulting strain was grown to saturation in YEPD_2%_ (10ml), collected by centrifugation, then transformed with a library of genomic fragments constructed from SEY6210 and cloned into the high copy number vector pRS424 (2μ). (This library was a gift from Dr. Howard Bussey, McGill University). Following a two hour recovery in YPD_2%_, the transformation mix was plated directly onto “petite media” [YEPE_3%_ supplemented with 0.1% dextrose (YEPE_3%_ + D_0.1%_)], and slow growth suppressors collected at a temperature of 30°C. In addition to obtaining multiple copies of *COQ7*, a single genomic fragment (28.57) containing *CLD1* was isolated.

### Cld1p homology model

A homology model of Cld1p was built remotely using the algorithm of Nielsen and colleagues [58] via the CPHmodels-3.0 server (*www*.*cbs*.*dtu*.*dk/services/CPHmodels/**)*. Epoxide hydrolase-1 from *Solanum tuberosum*, [59], (PDB structure 2CJP.A), served as the model-building template (alignment length 335 amino acids, double-sided Z-score 28.6). The catalytic triad of Cld1p (H424, D392, S230) was originally identified using STRAP [60], which facilitated the structure-guided alignment of several hundred α/β hydrolase fold-containing proteins, and the authenticity of this triad has now been confirmed experimentally [37].

### Western blotting

Yeast strains were cultured in YEPE_3%_ (3ml) at room temperature (RT, 25°C), with shaking (100 rpm). Cultures were harvested upon reaching stationary phase, the precise time depending on the particular strain. Cells were pelleted, resuspended in 200 μl boiling yeast lysis buffer (1% SDS, 62.5mM Tris-HCl, pH 6.8, 8M urea, 4% β-mercaptoethanol, 0.0125% bromophenol blue), boiled for two minutes, vortexed in the presence of ½ volume glass beads (5 mins), boiled for a further two minutes, then 20 μl of solubilized protein loaded onto a 4–20% Novagen pre-cast SDS-PAGE gel. Coq7p-HA was detected using western analysis after transfer to nitrocellulose (18V, 14 hr., RT) and incubation with a mouse, anti-haemagglutinin (HA) antibody (Babco, monoclonal 16B12, 1:500 dilution). Protein loading was quantified using Coomassie Blue.

### Total membrane lipid extraction

Total membrane lipids were isolated using a modification of the Bligh and Dyer method [61]. Briefly, relevant yeast strains were grown to an OD_600_ of 3.7, 10.5 and 13.2, then harvested by centrifugation (10 min at 624 × *g*, 4°C). A standardized cell-pellet wet weight of 1 gram was employed for all subsequent analyses. For spheroplasting, cell pellets were resuspended in ice cold water (20 ml), washed twice in the same volume of water, then resuspended in 1.4 ml/wet weight (g) pellet in pre-warmed spheroplast buffer (100 mM Tris-HCl (pH 9.3), 5 mM EDTA and 1% β-mercaptoethanol or 10 mM DTT), for 15 mins at 30°C. The cell suspension was then centrifuged at 4°C, washed thrice with 20 mM potassium phosphate buffer (pH 7.65), then the cell pellet resuspended in 20 mM potassium phosphate (pH 7.65) containing 1.2M sorbitol 4 ml/wet weight (g) pellet. Cells were then warmed to 40°C for 3–4 mins and Zymolyase (Fisher Scientific) added to a final concentration of 2.5 mg/g. The cell suspension was incubated at 30°C for 3 hours. At the end of the incubation, spheroplasts were pelleted by centrifugation, washed three times in 1.2M sorbitol (2 ml /wet weight (g)), then resuspended in 1 ml of the same solution. For each 1 ml sample, 3.75 ml of 1:2 (v/v) chloroform:methanol was added and samples vortexed for 2 minutes. Subsequently, 1.25 ml of chloroform and 1.25 ml of distilled water was also added. The bottom phase was recovered following centrifuging at 1500 x *g* for 5 mins. The extracted solvent was evaporated under air flow and then the lipid extract was re-dissolved in 50 μl of a 2:1 mix of chloroform/methanol (v/v). The lipids were then stored in glass vials with butylated hydroxytoluene (BHT) to prevent lipid oxidation, ready for separation by Thin Layer Chromatography (TLC).

### Thin layer chromatography

Analysis of cardiolipin and monolysocardiolipin in lipid extracts from whole-cell lysates and mitochondrial fractions was undertaken using TLC. Briefly, lipids were separated using Whatman^**®**^ Partisil™ LK5 TLC plates, following the methodology of Vaden and colleagues [62]. TLC plates were first wetted with 1.8% boric acid in ethanol, then dried at 110°C for 15 minutes to activate the silica. Next, equal volumes (10 μL) of test samples were spotted onto the relevant lane of a TLC plate then separated using a solvent phase consisting of 30:35:7:35 (v/v/v/v) chloroform/ethanol/water/triethylamine. When separation was complete, TLC plates were vacuum dried and spots developed using sulfuric acid charring. Spots were identified using purified lipid standards that included cardiolipin (Avanti polar lipids, # 840012P), phosphatidylserine (Avanti polar lipids, # 870336P), and a defined mixture of abundant eukaryotic lipids extracted from Soy (Avanti Polar Lipids, # 690050P). Developed spots were immediately photographed and then spot area and density quantified using Image J (http://imagej.nih.gov/ij/). For statistical testing, MLCL to CL ratios were calculated for samples measured in triplicate technical replicates, collected from duplicate independent experiments, at three separate OD_600_ values. Significance testing for strain- and growth phase-related differences was undertaken using multiple regression analysis (Real Statistics Resource Pack software, Release 4.3). Details provided in S2 Table).

### Q_6_ and DMQ_6_ quantification

Yeast quinone levels were quantified using a modification of the procedure described by Schultz and colleagues [63]. Strains of interest were cultured in liquid YEPE_3%_ at 25°C to the following optical densities (OD_600_, 1 cm pathlength): 1.7, 3.7, 6.7, 8.7, 10.5, 13.2. In total, three independent experimental replicates were collected for each OD_600_. Cells were collected by centrifugation (10 min at 624 × *g*, 4°C), washed once with H_2_O (10 ml), transferred to 50-ml pre-weighed glass tubes, then re-pelleted and again resuspended in H_2_O (12 ml). Three 1 ml aliquots were taken from each sample and cell pellet wet-weights determined. Cell pellets were dried for 1 hr. at 56°C and then their dry-weights determined. The average of the dry- to wet-weight ratio for the three 1 ml aliquots was then used to calculate the dry weight of the remaining sample. The remaining cell suspension was centrifuged, and the total wet weight of the cell pellet was determined. Glass beads (10 times the cell pellet wet weight), and 250 μl of Q_9_ internal standard (20 μg/ml) were added to the pellet and then the tubes were immediately flooded with nitrogen, capped, covered with foil, and kept on ice to prevent oxidation. Cells were lysed by vortexing for 2 min. Lipids were extracted by adding water/petroleum ether/methanol (1:4:6) and vortexing for an additional 30 sec. In some instances the water component was replace by 1M NaCl to enhance phase separation. Phases were separated by centrifugation (10 min at 624 × *g* and 4 °C). The upper petroleum ether layer was then transferred to a 10-ml glass tube. 4 ml of petroleum ether was added to the glass bead-aqueous phase, and the samples were re-vortexed for 30 sec. The petroleum ether layers from a total of three extractions were pooled and dried under nitrogen. Lipids were resuspended in a final volume of 1 ml isopropanol. Quinones (Q_6_, DMQ_6_ and Q_9_) were separated by reversed-phase high pressure liquid chromatography using a C18 column (Alltech Econosphere 5-μm, 4.6 × 250-mm, isocratic mode, mobile phase 72:20:8 (v/v/v) methanol/ethanol/propanol, 1 ml/min, 40°C) and quantitated using a Waters 600E UV detector at a wavelength of 275 nm. Peaks representing Q_6_ (Avanti polar lipids, # 900150O) and Q_9_ (Sigma, # 27597) were identified using purified standards. The peak representing DMQ_6_ was identified using a quinone extract from the yeast strain NM101, which is unable to manufacture Q_6_ and instead accumulates this intermediate [15]. Peak areas were normalized based on starting dry weight and then significance testing for quinone-, strain- and growth phase-related differences was undertaken using multiple regression analysis (Real Statistics Resource Pack software, Release 4.3). Details are provided in S3 Table.

### Replicative lifespan analysis

Replicative lifespan assays were conducted at 25°C following the procedure of Steffen and colleagues [64], except YEPE_3%_ + 2% agar was employed. Strains were coded to remove scoring bias. A total of 40 virgin cells (over replicate experiments) for each strain was analyzed using microdissection and the number of daughter cells that budded off from individual cells was recorded. Mother cells were monitored until no new buds were produced. Data was analyzed using a log rank test with significance (*p<0*.*05)* set against a chi square distribution using one degree of freedom (χ^2^ >3.84). Mother cells that were either damaged or could not be distinguished from daughter cells were censored from the analysis. Censored daughter cells were excluded from the calculation of mean RLS in Fig 7.

### High performance liquid chromatography—electrospray ionization—tandem mass spectrometry (HPLC-ESI-MS/MS)

Four or five cultures of relevant yeast strains were cultured at 25°C in YEPE_3%_ to mid log phase (OD_600_ 6–10). Sample OD_600_ values were adjusted to 7.0 and a 35 ml volume was then processed for HPLC-ESI-MS/MS as follows. Cells were collected by centrifugation (10 min at 624 × *g*, 4°C), washed with MS-grade H_2_O (3 x 10 ml), then cardiolipins (CLs) and monolysocardiolipins (MLCL) extracted from yeast pellets with methanol, chloroform, and 0.1N HCl (1:1:0.9) following the procedure of Bazan and colleagues [44]. Briefly, cell pellets were homogenized with a Mini-Beadbeater-24 Homogenizer (BioSpec Products). CL (14:0)_4_ [1',3'-bis[1,2-dimyristoyl-sn-glycero-3-phospho]-sn-glycerol] (Avanti Polar Lipids, #710332P) was added as the stable isotope-labeled standard prior to extraction. MS analyses were conducted on a Thermo Fisher *Q* Exactive (San Jose, CA) with on-line separation using a Thermo Fisher/Dionex RSLC nano HPLC system and Waters Atlantis dC18 column (150 μm x 105 mm; 3 μm) (Waters Corporation, Massachusetts). The gradient was started at 10% B and run from 10% B to 99% B over 40 min with the flow rate of 6 μl/min—where mobile phase A was acetonitrile/water (40:60) containing 10 mM ammonium acetate and mobile phase B was acetonitrile/isopropanol (10:90) containing 10 mM ammonium acetate. Data-dependent analyses were conducted using one full MS scan (70,000 resolution) followed by six tandem-MS scans with electrospray negative ion detection. Quantitative results were obtained by referencing experimental MLCL peak areas against a standard curve of CL(18:1)_4_ [1',3'-bis[1,2-dioleoyl-sn-glycero-3-phospho]-sn-glycerol] (Avanti Polar Lipids, #710335P), following CL(14:0)_4_ normalization. Significance testing for differences between strains was undertaken using Student’s *t-test (p < 0*.*05)*, without correction for multiple testing.

## Supporting Information

S1 Table *coq7* Hypomorphic Series. Detailed description of alleles comprising *coq7* hypomorphic series. (Corresponds to Table 1).
(XLS)

S2 Table Thin Layer Chromatography Regression Analysis. Variable re-coding used for multiple regression analysis. (Corresponds to Fig 4A).
(XLSX)

S3 Table DMQ_6_ and Q_6_ Regression Analysis. Variable re-coding used for multiple regression analysis. (Corresponds to Fig 6A–6F).
(XLSX)

S4 Table Raw Replicative Lifespan Data. (Corresponds to replicative life span data shown in Fig 7A and7B).
(XLSX)
